# Species delimitation of tea plants (*Camellia* sect. *Thea*) based on super-barcodes

**DOI:** 10.1186/s12870-024-04882-3

**Published:** 2024-03-11

**Authors:** Yinzi Jiang, Junbo Yang, Ryan A. Folk, Jianli Zhao, Jie Liu, Zhengshan He, Hua Peng, Shixiong Yang, Chunlei Xiang, Xiangqin Yu

**Affiliations:** 1 CAS Key Laboratory for Plant Diversity and Biogeography of East Asia, Kunming Institute of Botany, Chinese Academy of Sciences, Kunming, 650201 Yunnan China; 2 Germplasm Bank of Wild Species, Kunming Institute of Botany, Chinese Academy of Sciences, Kunming, 650201 Yunnan China; 3 Department of Biological Sciences, Mississippi State University, Starkville, 39762 MS USA; 4 Yunnan Key Laboratory of Plant Reproductive Adaptation and Evolutionary Ecology, Laboratory of Ecology and Evolutionary Biology, School of Ecology and Environmental Sciences, Yunnan University, Kunming, 650500 Yunnan China

**Keywords:** Tea plants, Plastome, Super-barcode, De novo species delimitation

## Abstract

**Background:**

The era of high throughput sequencing offers new paths to identifying species boundaries that are complementary to traditional morphology-based delimitations. De novo species delimitation using traditional or DNA super-barcodes serve as efficient approaches to recognizing putative species (molecular operational taxonomic units, MOTUs). Tea plants (*Camellia* sect. *Thea*) form a group of morphologically similar species with significant economic value, providing the raw material for tea, which is the most popular nonalcoholic caffeine-containing beverage in the world. Taxonomic challenges have arisen from vague species boundaries in this group.

**Results:**

Based on the most comprehensive sampling of *C.* sect. *Thea* by far (165 individuals of 39 morphospecies), we applied three de novo species delimitation methods (ASAP, PTP, and mPTP) using plastome data to provide an independent evaluation of morphology-based species boundaries in tea plants. Comparing MOTU partitions with morphospecies, we particularly tested the congruence of MOTUs resulting from different methods. We recognized 28 consensus MOTUs within *C.* sect. *Thea*, while tentatively suggesting that 11 morphospecies be discarded. Ten of the 28 consensus MOTUs were uncovered as morphospecies complexes in need of further study integrating other evidence. Our results also showed a strong imbalance among the analyzed MOTUs in terms of the number of molecular diagnostic characters.

**Conclusion:**

This study serves as a solid step forward for recognizing the underlying species boundaries of tea plants, providing a needed evidence-based framework for the utilization and conservation of this economically important plant group.

**Supplementary Information:**

The online version contains supplementary material available at 10.1186/s12870-024-04882-3.

## Introduction

While DNA sequences and other kinds of integrative data are increasingly included in the assessment of species boundaries, species delimitation in plants generally remains defined on the basis of morphological characters [1–3]. Morphology-based species delimitation is contentious for two reasons. First, intra- and inter-specific variation among closely related taxa often overlaps, commonly including morphological traits that have been used to distinguish taxa [4]. Second, different taxonomists may disagree with each other on the taxonomic significance of the same morphological trait [5]. It is quite common that the same specimens, especially of taxonomically difficult taxa, are assigned different names by different taxonomists, or even by the same taxonomist at different times. Thus, the application of molecular sequence data in delimiting species is a useful adjunct for resolving groups in which morphological data are indecisive, as well as for identifying currently unrecognized species-level diversity [6, 7], which in both cases may reciprocally enhance the application of morphological data [8]. Finally, accelerating the pace of taxonomic work is urgently needed to meet the challenge of the contemporary biodiversity crisis in light of climate change and anthropogenic alteration. For these and other reasons, DNA-based species delimitation and identification has been proposed for and attracted substantial interest as a complement to morphology-based taxonomy [9–11]. For this purpose, DNA barcoding techniques espouse the use of homologous DNA fragments applicable across relatively wide phylogenetic scales to identify or delimit taxa [12–15].

Currently, many empirical studies have investigated the robustness of DNA barcodes in identifying species under the prior knowledge of species identification based on morphology [16–20]. However, for highly morphologically similar taxa, taxonomic discordances between these and morphological identifications are quite common [5]. Operational factors involved in discordance include species over-splitting and lumping, which can significantly affect evaluations of the efficacy of DNA barcodes [20, 21]. In the light of this, de novo molecular species delimitation using DNA barcodes was therefore advocated without prior biological assignments [10, 11]. Originally, DNA barcodes were short DNA fragments selected from organellar and nuclear genomes, such as COI, *rbcL*, *matK*, *trnH–psbA* and nrITS. In the last decade, super-barcode data from the entire plastome have become widely used in identifying species [14, 22, 23], where their greater signal has found use in several taxa. For example, molecular delimitation in *Orychophragmus* (Brassicaceae) highlighted the application of plastomes to jointly examining species boundaries and establishing phylogenetic relationships [24]. Another case based on *Polygonatum kingianum* demonstrated the ability to delimit species on the basis of the plastid genome [25].

Tea plants generally refer to plants of *Camellia* sect. *Thea* (Theaceae). Almost all of the species from this section can be prepared as a nonalcoholic caffeine-containing beverage [26, 27]*.* The most commonly and commercially grown tea plants are *C. sinensis* var. *sinensis* and *C. sinensis* var. *assamica* [26], but other closely related species are potential beverage resources that need further investigation. Further work on potential uses of tea plants is hampered by a complex and controversial taxonomic history. There are three important taxonomic systems of *C.* sect. *Thea.* Sealy [28] first systematically studied genus *Camellia* in modern times and he proposed classification system of it, where only five species were included in *C.* sect. *Thea.* However, with more *Camellia* species being discovered, Sealy’s classification system was gradually replaced by the other two classification systems which are widely used now. One is Chang’s taxonomic system; Chang [29] recognized thirty-two species in four series of *C.* sect. *Thea*. The other is Ming’s taxonomic system; Ming [30] recognized twelve species without establishing any series in *C.* sect. *Thea*. The dramatic variation between these two taxonomic treatments for *C.* sect. *Thea* is due primarily to emphasizing different morphological characters. The existence of these significant differences, leading to conflicting morphology-based classifications, has made traditional taxonomic work and investigation of wild tea relatives in *C.* sect. *Thea* intractable. Additionally, new species have continued to be found since these treatments [31–35]. Since the publication of the first classification system for *C.* sect. *Thea* (1958), the recognized species diversity of tea plants has increased significantly (up to 54 new taxa published), mainly through in-depth field investigation and analysis of morphological characters [27]. However, this progress leads to doubts on the true level of species diversity of tea plants and confusion on the protection and efficient utilization of tea plants. Significantly, all species of *C.* sect. *Thea* are listed as protected in the updated List of National Key Protected Wild Plants of China in 2021 and are ranked as category II, while the numbers of species are not specified [36]. Therefore, understanding species diversity and properly delimiting species boundaries are of realistic value in both conserving and utilizing tea plants.

**Fig. 1:**
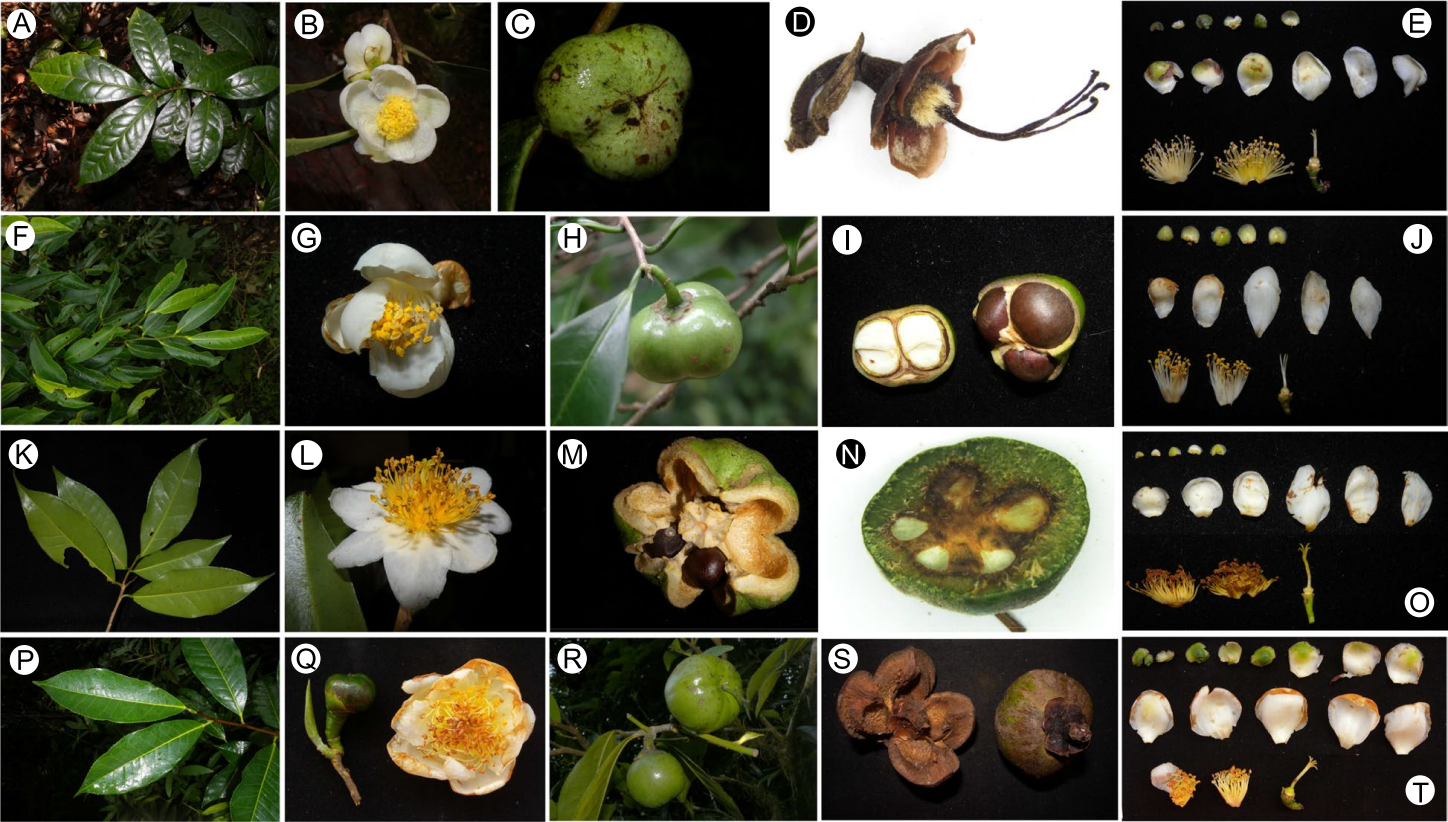
Selected species of *Camellia* sect. *Thea*, illustrating the morphological diversity of tea plants. **A**-**E**: *C. sinensis* var. *sinensis*; **F**-**J**: *C. costata*; **K**–**O**: *C. taliensis*; **P**–**T**: *C. kwangsiensis*

Based on our careful examination of specimens and wide-range field survey, most tea plants are highly morphologically similar, with small differences in ovary, sepal, and the size of flowers and fruits being the primary morphological variation (Fig. 1). Notably, in some of the diagnostic morphological characters variation often overlaps, making species identification and delimitation in tea plants extremely challenging and also leading to a misestimate of the species diversity of tea plants. Despite the disagreements on the classification of tea plants, previous phylogenetic studies have primarily tested the monophyly of *C.* sect. *Thea* [37–39] without further detailed study on species delimitation within the section. Molecular species delimitation using DNA barcodes has not yet been applied in *C.* sect. *Thea*, with few studies only focusing specifically on *C. sinensis* that did not further discuss the species delimitation of the section due to limited sampling [34, 40].

Currently, plastomes can be rapidly and inexpensively obtained due to the improvement of next-generation sequencing (NGS) techniques [41–43]. Here, we collected 165 samples of major extant species of *C.* sect. *Thea* and DNA barcoded by sequencing plastomes. Based on multiple molecular species delimitation approaches, we evaluated the effectivity of the plastome as a super-barcode in delimiting tea plants. We aim to reassess the validity of morphology-based species boundaries of tea plants using plastomes, and to explore the underlying species-level diversity of tea plants.

## Materials and methods

### Sample collection and sequencing

**Fig. 2:**
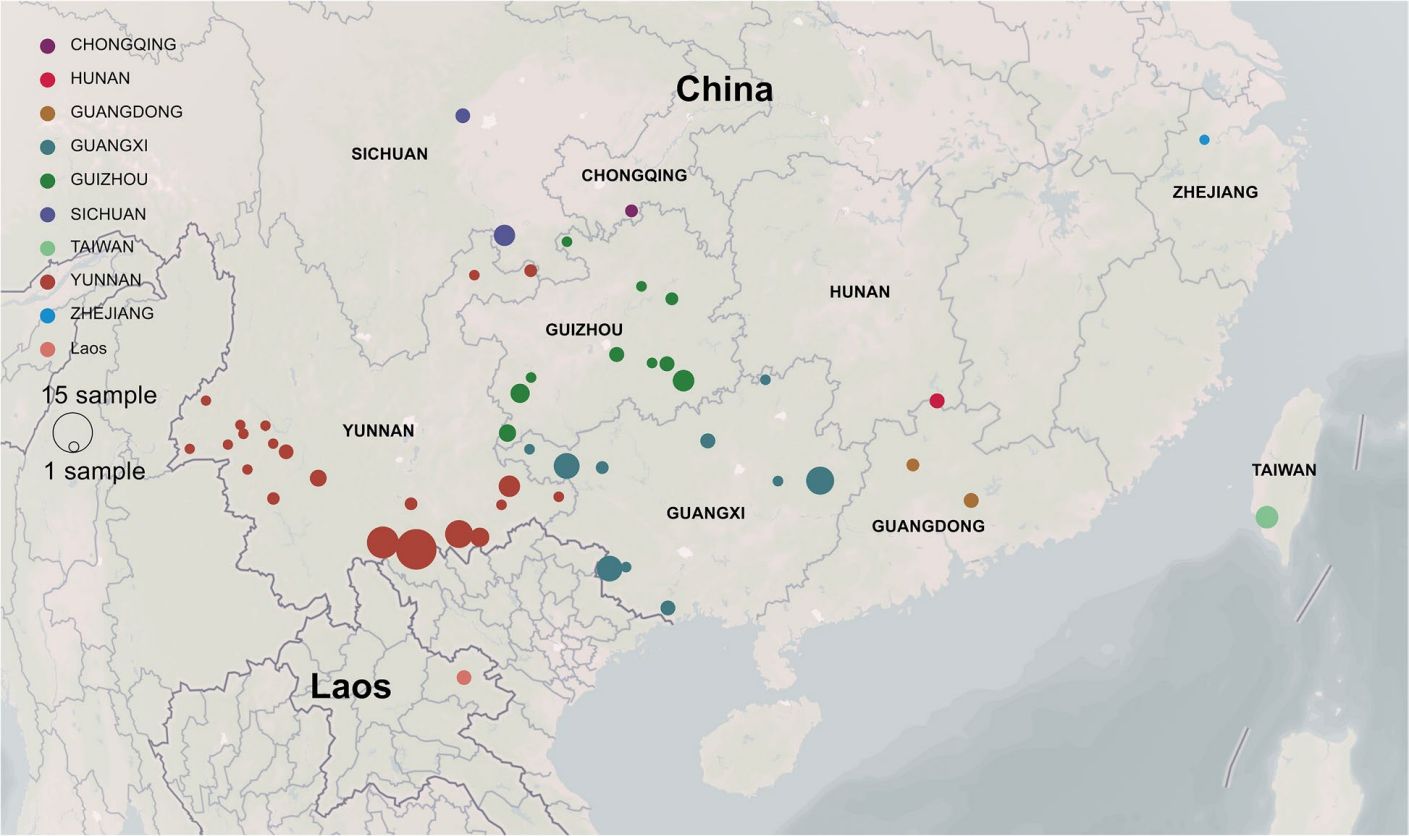
Map of geographical distribution of samples of *C.* sect. *Thea* for this study. The map was constructed using the mapbox (https://studio.mapbox.com/)

The collected samples were assigned to morphospecies by reference to recent taxonomic literature. Both the Chang and Ming’s classification systems were integrated in this study [29, 30]. Herbarium material (including types) for each species was also investigated, and most samples were collected from type localities. Dr. Shixiong Yang undertook the formal identification of the plant material used in our study. All samples were collected in the field (Fig. 2) with fresh leaf tissue dried in silica gel. In total, plastomes of 165 samples representing 39 morphospecies of *C.* sect. *Thea* were sequenced (Table S1), of which 34 morphospecies were represented by more than one individual (2–10) and five morphospecies were singletons (population-level sampling in Table S1). *Camellia mairei* (GenBank accession: KY406767) and *C. reticulata* (GenBank accession: KY406793) from *C.* sect. *Camellia* were selected as outgroups, whose plastome data were obtained from our previous study [44]. The vouchers were deposited in the Herbarium of the Kunming Institute of Botany (KUN), Chinese Academy of Sciences, Yunnan, China.

Dried samples were then subjected to total genomic DNA extraction following a modified cetrimonium bromide (CTAB) protocol [45], followed by two alternative sequencing methods. For 115 samples, the plastomes were amplified in overlapping fragments using the long-range PCR method [46], and PCR fragments were then pooled together in roughly equal concentrations for subsequent sequencing. Paired-end sequencing of 250 bp was conducted on the Illumina MiSeq platform at the Molecular Biology Experiment Center, Germplasm Bank of Wild Species in Southwest China. About 200 Mb − 2Gb sequencing data were generated for each sample. For the genome skimming of the remaining 50 samples, paired-end sequencing libraries were generated from total DNA following the manufacturer’s protocol (Illumina) with an insertion size of 350 bp and were sequenced on the Illumina NovaSeq 6000 platform with the 150-bp paired-end sequencing model. Approximately 2 Gb sequencing data were generated for each sample.

### Genome assembly, annotation, and alignment

Quality control of raw sequence reads was performed using fastp v0.20.1 [47] with default parameters. Plastomes were first de novo assembled using GetOrangelle pipeline v1.7.5.2 [48] based on the reads from both long-range PCR and genome skimming sequencing. Successfully completed assemblies were annotated using PGA [49], then the intron/exon boundaries were manually checked and adjusted. Most reads generated by long-range PCR method failed to be assembled as a circle plastome using GetOrganelle pipeline v1.7.5.2 [48] and only contigs were obtained. Therefore, for these samples, we used a reference sequence (*Camellia remotiserrata*; GenBank accession number KY406759) as a mapping reference to complete the assembly. Contigs from long-range PCR reads were mapped to the reference using Bowtie 2 [50] with default parameters. The obtained mapping files were exported in SAM (Sequence Alignment/Map) format. Next, using Geneious v8.02 [51], consensus sequences were extracted from these SAM files and then aligned using MAFFT plugin with default parameters, then manually checked and adjusted. The consensus sequences were annotated according to the reference. Including two outgroups, plastome sequences (excluding one IR region) from 165 individuals were aligned using MAFFT v7.471 [52] with default settings. Poorly aligned regions were refined by trimAl v1.4.rev15 [53] using "-automated1" command, then manually checked and adjusted.

### Genetic diversity analysis

Nucleotide diversity across 165 samples was estimated using DnaSP v6.12.03 [54] with a sliding window of 2,000 bp and step size of 200 bp. The maximum intraspecific genetic distances among 39 morphospecies were calculated using MEGA X [55]. Analyses were conducted using the Kimura 2-parameter model [56].

### Phylogenetic analysis

Maximum likelihood (ML) analysis was performed using RAxML v8.2.12 [57] based on the General Time Reversible + gamma model (GTR-GAMMA model) with 1000 rapid bootstraps replications. Bayesian inference (BI) analysis was performed using MrBayes v3.2 [58]. Two independent Markov chain Monte Carlo (MCMC) runs were executed. Four chains were run for two million generations with random initial trees and sampling every 100 generations. The first 25% of the trees were discarded as burn-in. Moreover, we carried out Quartet Sampling (QS) analysis based on plastome dataset and the ML tree with 1,000 replicates to dissect phylogenetic discordance within plastome, which were recently found in other organisms [59–62]. The QS method was designed to evaluate the consistency of information (Quartet Concordance score, QC), the presence of secondary evolutionary histories (Quartet Differential score, QD), the amount of information (Quartet Informativeness score, QI), and the reliability of individual taxa in the tree (Quartet Fidelity score, QF). The resultant trees were visualized and edited in FigTree v1.4.4 [63].

### Molecular species delimitation analysis

Two methods (tree-based and distance-based methods) were used for de novo species delimitation of *C.* sect *Thea* based on plastome sequences. The above two approaches were performed using the PTP (Poisson Tree Processes) model [64], and ASAP (Assemble Species by Automatic Partitioning) [65], respectively. The PTP model is a tree-based method that models the expected number of substitutions for intra- vs. interspecific gene tree branch lengths, identifying transition points in the tree as delimited species [64]. The PTP modeling was performed with PTP web server (https://species.h-its.org/) with 500,000 replicates using the maximum likelihood implementation (PTP-ML), as well as the Bayesian implementation (PTP-BI). In addition, multirate PTP (mPTP) was performed to further assess the confidence of the previous PTP analyses by accounting for differences between species in sampling and genetic structure [66]. The ML tree was used as input for all PTP analyses.

In contrast, distance-based methods do not utilize the tree topology, but instead rely on genetic distances, such as the ASAP algorithm [65]. ASAP analysis was conducted on the webserver (https://bioinfo.mnhn.fr/abi/public/asap/) based on *p* distance model using the plastome alignment. The ASAP algorithm partitions sequence into “group” by ascending hierarchical clustering based on sequence similarity [65]. The best two MOTUs partition predicted by ASAP were chosen to compare with other methods.

### Comparison of morphospecies and MOTU

The congruence between MOTUs and morphospecies assignment was evaluated by the match ratio (morphology) [67]. The match ratio (morphology) is equal to 2 × N_morph_/(N_MOTU_ + N_MORPH_), where N_morph_ is the number of matches of morphospecies (all samples) with MOTUs, N_MOTU_ is the number of MOTUs, and N_MORPH_ is the number of morphospecies. Since nonmonophyletic species are unlikely to be correctly delimited by any method based on molecular data [68–70], for operational purposes, we recognized monophyletic morphospecies based on the ML tree. The congruence among different molecular species delimitation methods were then assessed by the match ratio (monophyly). The match ratio (monophyly) is equal to N_mono_/N_MONO,_ where N_mono_ is the number of MOTUs that were consistent with monophyletic morphospecies, and N_MONO_ is the number of total monophyletic morphospecies.

Finally, to account for uncertainty and limitations in the implementation of individual methods [71], consensus MOTUs (c-MOTUs) were determined following several criteria [70, 72]: (i) MOTUs that were delimited identically by two of three methods were accepted; (ii) the c-MOTUs were monophyletic; (iii) sympatric MOTUs were accepted while allopatric MOTUs were rejected. We thereafter calculated the molecular diagnostic characters (MDC) for each c-MOTU using FASTACHAR v. 0.2.5 software [73].

## Results

### Sampling, characteristics of sequencing data and datasets

For the newly sequenced 165 samples, the number of cleaned Illumina sequencing reads ranged from 251,240 to 30,000,000. The de novo assembly generated 165 complete or near-complete plastomes, ranged from 130,314 to 133,043 bp when excluding one IR region (Table S1). The aligned dataset of plastome sequences was 131,047 bp after manual adjustment. It included 2,183 variable sites (1.67%), among which 1,771 sites were parsimony-informative (1.35%). Based on the DNA polymorphism analysis, we found that the plastome variability was very low with an overall nucleotide diversity (0.00146) ≤ 0.01 (Table 1).

**Table 1:** Summary statistics of the number of individuals per morphospecies (N), the number of collection sites per morphospecies (N_c_) and the maximum intraspecies genetic distance (D)

Mophospecies	N	Nc	D
*Camellia arborescens*	2	2	0.001703
*Camellia atrothea*	3	1	0.000076
*Camellia changningensis*	1	1	NA
*Camellia costata*	8	1	0.000704
*Camellia crispula*	7	2	0.000752
*Camellia crassicolumna* var. *shangbaensis*	3	1	0.000008
*Camellia danzaiensis*	3	1	0.000299
*Camellia dishiensis*	2	1	0.000023
*Camellia fangchengensis*	3	1	0.000000
*Camellia formosensis*	7	1	0.000490
*Camellia glaberrima*	3	1	0.000061
*Camellia grandibracteata*	3	1	0.000054
*Camellia gymnogyna*	3	1	0.000283
*Camellia haaniensis*	4	1	0.000054
*Camellia kwangnanica*	5	2	0.000612
*Camellia kwangsiensis*	7	2	0.001779
*Camellia kwangtungensis*	2	1	0.000069
*Camellia leptophylla*	6	1	0.001854
*Camellia longlingensis*	1	1	NA
*Camellia makuanica*	5	1	0.000115
*Camellia multiplex*	6	3	0.000872
*Camellia nanchuanica*	2	1	0.000046
*Camellia parvisepala*	2	1	0.000000
*Camellia parvisepaloides*	1	1	NA
*Camellia pentastyla*	1	1	NA
*Camellia polyneura*	4	1	0.000046
*Camellia ptilophylla*	3	1	0.000505
*Camellia pubescens*	3	1	0.000031
*Camellia quinquelocularis*	3	1	0.000008
*Camellia remotiserrata*	8	3	0.000697
*Camellia sinensis* var. *sinensis*	6	5	0.000038
*Camellia sinensis* var. *assamica*	10	4	0.000582
*Camellia sinensis* var. *kucha*	5	1	0.000882
*Camellia sinensis* var. *dehungensis*	1	1	NA
*Camellia sinensis* var. *pubilimba*	12	7	0.001926
*Camellia tachangensis*	5	2	0.001779
*Camellia taliensis*	5	5	0.000898
*Camellia tetracarpa*	5	1	0.001748
*Camellia yungkiangensis*	6	1	0.000499

### Phylogenetic reconstruction

**Fig. 3:**
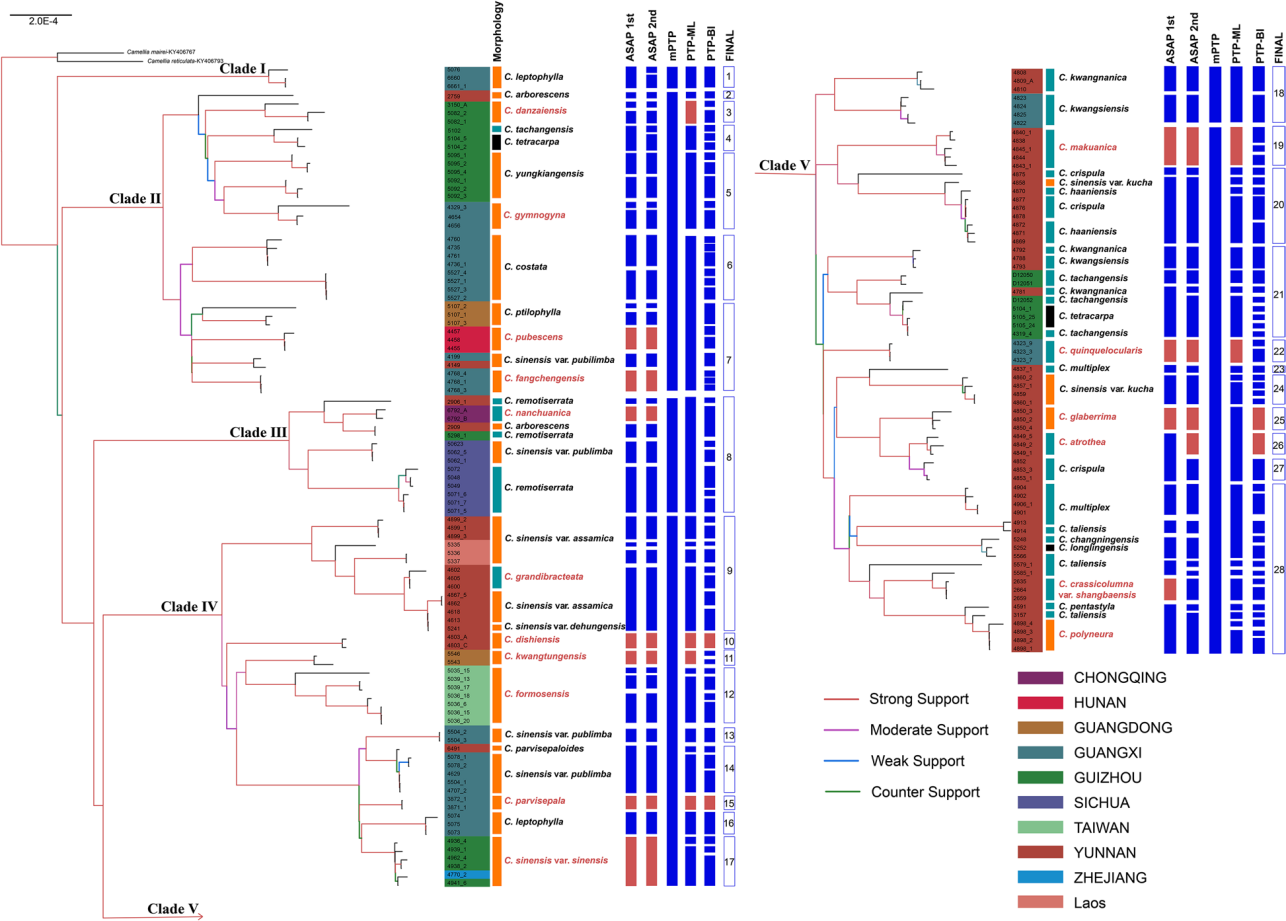
Maximum likelihood phylogeny of tea plants with information of morphospecies assignments, information of collection sites, and delimited MOTUs. The branch colors represent the QS scores (QC/QD/QI). Red represents full or strong support (QC ≥ 0.5), purple represents moderate support (0.2 ≤ QC < 0.5), blue represents weak support (0 ≤ QC < 0.2), and green represents counter support (QC < 0), according to Pease et al. (2018). The geographical distribution of collection sites is marked on the voucher number (sample name) with different colors. Taxa names in red indicate monophyly and taxa names in black indicate non-monophyly. Red solid boxes besides taxa names indicate agreement between molecular species delimitation method and morphospecies assignment, while blue solid boxes indicate disagreement. Hollow boxes indicate the final MOTUs

Phylogenetic reconstructions based on RAxML and MrBayes analyses obtained identical topology (Fig. S1), and five clades were identified (Fig. 3). Seventeen morphospecies (43.59%) were recovered as monophyletic, including *Camellia atrothea*, *C. crassicolumna* var. *shangbaensis*, *C. danzaiensis*, *C. dishiensis*, *C. fangchengensis*, *C. formosensis*, *C. glaberrima*, *C. grandibracteata*, *C. gymnogyna*, *C. kwangtungenesis*, *C. makuanica*, *C. nanchuanica*, *C. parvisepala*, *C. polyneura*, *C. pubescens*, *C. quinquelocularis*, and *C. sinensis* var. *sinensis* (Fig. 3).

Clade I consisted of three individuals of *C. leptophylla* while other three individuals formed a subclade clustered within Clade IV, although all of them were collected from the type locality (Longzhou, Guangxi, China). Clade II contained 11 morphospecies, all samples of *C. danzaiensis*, *C. pubescens*, *C. gymnogyna* and *C. fangchengensis* clustered as monophyletic groups, respectively. Most morphospecies in this clade were characterized by having 3-locule ovary except for *C. tachangensis* and *C. tetracarpa*.

Clade III exclusively contained *C. remotiserrata* (2906, 5298, 5048, 5049, 5071, 5072) and *C. nanchuanica* (6792), as well as cultivated individuals of *C. arborescens* (2909) and *C. sinensis* var. *pubilimba* (5062), both of which were introduced by wild *C. remotiserrata* germplasm. Species in this clade exhibited a geographic cluster pattern. Sample 2906 of *C. remotiserrata*, 2909 of *C. arborescens* (from Yunnan, Weixin), 5048, 5049, 5071, 5072 of *C. remotiserrata* (from Sichuan, Yibin), 5298 of *C. remotiserrata* (from Guizhou Chishui) and 6792 of *C. nanchuanica* (from Chongqing Nanchuan) overlapped in distribution.

Clade IV included 10 morphospecies, of which six were resolved as monophyletic. Most samples of *C. sinensis* (*C. sinensis* var. *sinensis*, *C. sinensis* var. *assamica*, *C. sinensis* var. *dehungensis* and *C. sinensis* var. *publimba*) were nested in this clade, along with their relatives (*C. dishiensis*, *C. formosensis*, *C. parvisepaloides*, *C. parvisepala*, and *C. grandibracteata*). Among the five relatives, except *C. grandibracteata*, four species have been synonymized under the name of *C. sinensis*. Samples of *C. kwangtungensis*, which has been put in *C.* sect. *Glaberrima* by Chang & Ren (1998), formed a monophyletic group. Clade V was the largest clade mainly composed of morphospecies with 5-locule ovary, five morphospecies were exceptions. *Camellia glaberrima*, *C. polyneura*, and *C. sinensis* var. *kucha* were characterized by having 3-locule ovary. *Camellia tetracarpa* had 4-locule ovary, and *C. longlingensis* varied from 3-locule to 5-locule ovary.

In the QS analysis, we considered a QC score of ≥ 0.5 to be strong support. In general, strong-supported nodes had long internal branches while low QC values were coupled with short branch lengths (Fig. 3). The mean QC score for the internodes was 0.65. Weak support (QC = 0.039) was found at the branch separating Clade II from the rest of samples (Fig. S2). All the monophyletic morphospecies were recovered with strong support (QC = 1). Moreover, branches with negative QC scores with high QD scores were observed in both 5-loculed and trilocular taxa, suggestive of putative incomplete lineage sorting (ILS) (Fig. S2). The average QI score for nodes was 0.86, and the majority (67%) were above the average, indicating that sequence variation was not a limiting issue. The QF scores for all samples were above 0.5, and the mean QF score was 0.77, indicating that rogue taxa were not an issue affecting phylogenetic inference (Table S2).

### Molecular species delimitation

The MOTUs produced by different molecular species delimitation methods conflicted with each other, as well as with the morphology-based species assignments (Table 2). PTP analysis resulted in two MOTU partitioning schemes. One was the maximum likelihood solution (PTP-ML), distinguishing 45 MOTUs with six matches and the match ratios were 0.14 for morphology and 0.35 for monophyly. The other was the Bayesian solution (PTP-BI); PTP-BI produced 100 MOTUs with five matches with the morphology (0.07) and monophyly (0.29). However, mPTP only produced six MOTUs and none of the MOTUs defined by mPTP were congruent with morphospecies. The best two ASAP results produced 54 or 61 MOTUs, thus the resultant match ratios with morphology were 0.26 and 0.21, respectively. Twelve monophyletic morphospecies (0.71) were both recovered in the two ASAP results. In total, 22 non-monophyletic morphospecies were inconsistent with MOTUs delimited by all methods. Additionally, not all monophyletic morphospecies agreed with MOTUs.

**Table 2:** Match ratio of molecular species delimitation methods on tea plants based on the congruence between MOTUs and morphospecies

	ASAP 1st	ASAP 2nd	PTP-ML	PTP-BI	mPTP
N match	12	12	6	5	0
N monophyly	12	12	6	5	0
N MOTU	54	61	45	100	6
Match Ratio (morphology)	0.26	0.24	0.14	0.07	0.00
Match Ratio (monophyly)	0.71	0.71	0.35	0.29	0.00

A final count of 28 c-MOTUs was therefore suggested. Among these, 16 c-MOTUs were detected by multiple conspecific samples, including c-MOTU-1, 3, 6, 10–13, 15–17, 19, 22, 24–27. Two c-MOTUs were consisted of singletons (c-MOTU-2 and -23). The other 10 c-MOTUs comprised morphospecies complexes, which appeared to be indistinguishable either by morphology or by plastomes. In total, we detected from 11 to 1,252 plastid MDCs among these 28 c-MOTUs. The lowest number of MDCs was found between c-MOTU-11 and c-MOTU-18. The highest number of MDCs was found between c-MOTU-4 and c-MOTU-19 (Table S3).

## Discussion

### Comparing the performance of molecular species delimitation methods

This study describes a protocol for rapidly obtaining a primary species delimitation scheme. In some cases, plastome data contributes to discovering cryptic diversity or sorting out problematic taxonomic treatments [24, 74, 75], and have formed the primary basis of formal taxonomic revisions [25, 76]. In other cases, an integrative strategy including plastome data has been applied for delimiting species. For example, species boundaries in the *Maddenia* group of *Prunus* were clarified based on not only plastomes but also nuclear data and morphology [77]. Compared with the varying steps needed for different types of data, our protocol has the advantage of using highly accessible single-locus data with defined criteria to produce a testable primary taxonomic framework.

The high incongruences between MOTUs and morphospecies in *Camellia* sect. *Thea*, together with the observed conflicts within different molecular species delimitation methods indicate that plastomes are unable to completely clarify morphology-based species boundaries of tea plants. The incongruence across delimitation methods is inevitable and attributable to the inherent limitations of methods [65, 78]. Successful applications of molecular species delimitation methods require intellectual and methodological consensus [71]. Different statistical approaches and a priori criteria for delimiting species might result in conflicting delimitations of species boundaries [79]. On the one hand, distance-based methods (e.g. ASAP) are based on a similarity criterion, while tree-based methods (e.g. PTP and mPTP) are based on a phylogenetic topology criterion. In addition, although PTP and ASAP are based on different criteria, they both agree on the premise that all species are expected to be reciprocally monophyletic, which accounts for the increased match rate when only considering monophyletic morphospecies in the study. However, many studies have found that non-monophyly is quite extensive among plant species [80–82]. Therefore, these criteria can be practically problematic to apply in delimiting species boundaries given the complex reticulation process in plants, such as introgression and hybridization [83, 84]. This is particularly true for tea plants, where the evolutionary history is further complicated by human intervention, such as describing new taxa from cultivation, and hybridization among tea plants when they are planted together [27, 85, 86].

On the other hand, sampling may also affect the output of different delimitation methods [67, 87, 88], as is the case of tea plants studied here. The discordance between monophyletic morphospecies and MOTUs indicated limited sampling could lead to monophyly ascertainment biases [67, 70]. In addition, oversampling of closely related individuals within one species might risk causing grouping some of individuals within other relatively distinct species, resulting in over-splitting the former species using molecular delimitation methods [89]. In two cases (*C. tetracarpa* and *C. tachangensis*), despite being sampled from the same locality, some of their samples nested with morphological distinct species, such as trilocular *C. costata* and *C. yungkiangensis*, while the other nested together. As expected, all the methods over-split them, hence violating the prior morphological assignments. In addition, geographic sampling bias may exacerbate intraspecific variation, which has been shown to decrease the efficacy of species delimitation using molecular data [64, 66]. In such cases, mPTP may be more accurate as it can account for divergent intraspecific variation among species [66, 78]. However, the estimations yielded by mPTP tend to be too conservative in empirical studies [90]. As exemplified for Clades II to V, mPTP collapsed many morphologically distinct taxa, some of which are uncontroversial, into one. However, the other methods likewise perhaps over-split compared with the mPTP result. This was observed in groups of monophyletic *C. formosensis*, which were delimited as several discrete MOTUs by PTP-ML, PTP-BI, and ASAP (Fig. 3). Therefore, considering that the species estimations yielded by these methods are likely the result of their inherent limitations, operational tendency to over-split or lump, and the general difficulty of species identification in *Camellia* sect. *Thea*, we propose using multiple methods to cross-validate with each other.

### Uncovering the species delimitation of tea plants

Our study sampled 39 morphospecies of tea plants, accounting for 75% of 52 legal names in *C.* sect. *Thea* under the Botanical Code [27, 91]. This therefore represents by far the most comprehensive sampling across *C.* sect. *Thea* attempted to date. The examined 39 morphospecies of tea plants resulted in 28 c-MOTUs considering the results among methods employed, which reflect those lineages likely to be distinct species. The final c-MOTUs were more than twice as numerous as those recognized in Ming’s taxonomic system while less numerous than Chang’s taxonomic system for *C.* sect. *Thea*. The introduction of molecular data, not always agreeing with morphological data, has therefore largely complicated the taxonomy of *C.* sect. *Thea.* In general, two interpretations can be drawn from the cases of incongruence between MOTU and morphospecies. One is that misleading morphological variation results in incorrect species delimitation. In these cases, incongruent MOTUs might instead reflect true species boundaries. The other is that molecular species delimitation methods are not powerful enough to solve complex biological factors underlying the speciation process because of limitations in algorithms or data.

We found conditions in tea plants were even more complex as the two interpretations appear to be interleaved. Ten morphospecies were merged with their sister morphospecies in a single c-MOTU partition (c-MOTU-4, 5, 7, 8, 9, 14, 18, 20, 21, and 28). In one case, *C. yungkiangensis* clustered with *C. gymnogyna* within one MOTU. However, the merge of *C. yungkiangensis* and *C. gymnogyna* was not supported by morphology. Similarly, Ming (1992) treated *C. yungkiangensis* as a synonym of *C. costata* [92], but *C. yungkiangensis* and *C. costata* nested in different subclades in Clade II in this analysis. Two morphospecies (*C. grandibracteata* and *C. haaniensis*) showed similar patterns: *C. grandibracteata* was merged with *C. sinensis* var. *assamica* and *C. haaniensis* was merged with *C. crispula* (Fig. 3). *C. grandibracteata* might be a hybrid of *C. sinensis* var. *assamica* and *C. taliensis*, which was also supported by morphological evidence [86]. In addition, *Camellia haaniensis* was synonymized under *C. crispula* [93]. In another case, *C. remotiserrata* and *C. nanchuanica* were merged in Clade III. First, Ming (1992) synonymized *C. remotiserrata* and *C. nanchuanica* under *C. gymnogyna* var. *remotiserrata* [92]. Later, Ming (1999) made a new combination—*C. tachangensis* var. *remotiserrata*, and therefore *C. remotiserrata* and *C. nanchuanica* became synonyms of *C. tachangensis* var. *remotiserrata* [94]. Our results supported that *C. remotiserrata* and *C. nanchuanica* should be the same species. However, the relationship between *C. remotiserrata* and *C. tachangensis* needs further investigation.

Considering the contrasting case where molecular data recognize more taxa, up to 10 morphospecies of *C.* sect. *Thea* were split into different MOTUs, including *C. leptophylla*, *C. arborescens*, *C. tachangensis*, *C. tetracarpa*, *C. sinensis* var. *pubilimba*, *C. kwangsiensis*, *C. kwangnanica*, *C. crispula*, *C. sinensis* var. *kucha*, and *C. multiplex*. The splits of 10 morphospecies were primarily associated with geographical clustering of individuals (in the same place or nearby), except for *C. leptophylla* and *C. sinensis* var. *pubilimba*. Therefore, these cases of splits might reflect the possibility of cryptic species.

Finally, 10 c-MOTUs agreed with morphology assignments: *Camellia atrothea*, *C. danzaiensis*, *C. dishiensis*, *C. formosensis*, *C. kwangtungensis*, *C. glaberrima*, *C. makuanica*, *C. parvisepala*, *C. quinquelocularis*, and *C. sinensis* var. *sinensis*. They might be distinct species but still need further validation. For example, *Camellia danzaiensis*, *C. glaberrima* and *C. kwangtungensis* belong to *C.* sect. *Glaberrima*, which is morphologically distinct with *C.* sect. *Thea* in Chang’s classification system [29]. However, Ming merged *C.* sect. *Glaberrima* into *C.* sect. *Thea* [92]. In addition, none of the three species were accepted as distinct species in Ming’s classification, in which *Camellia danzaiensis* and *C. kwangtungensis* were synonymized under *C. costata* and *C. glaberrima* was synonymized under *C. gymnogyna* [92, 94]. Our phylogenetic results indicated that three species of sect. *Glaberrima* had a close relationship with species of *C.* sect. *Thea*. In addition, all three species were resolved as monophyletic groups and therefore recovered as three c-MOTUs, respectively.

According to botanical codes of nomenclature, scientific names are based on type specimens, which are often not available for DNA analysis, or in some cases are even lost. Therefore, in assemblages of taxonomically difficult morphospecies, species names associated with molecular diagnostic characters tend to have greater taxonomic utility in many contexts than those solely based on morphology.

## Conclusion

Our study integrated multiple species delimitation approaches based on plastome data to evaluate the validity of 39 morphospecies in *C.* sect. *Thea*. We proposed 28 c-MOTUs, fewer than species assignments based on morphology. Although, the number of molecular diagnostic characters varied irregularly among 28 c-MOTUs. The fact that molecular species delimitation of tea plants conflicts with morphology highlights the incompatibility of extant taxonomic systems of *C.* sect. *Thea*. Even without accurate prior biological knowledge, an estimate of species richness and delimitation can be obtained through the simple and fast algorithmic processing of molecular data. While taxonomic decisions based on analyses of plastome data do pose risks, they are useful if the results of molecular species delimitation are viewed as drafts for taxonomy rather than as the sole criterion for species description. Therefore, our research provides taxonomists with a starting point for taxonomic revision of *C.* sect. *Thea*.

### Supplementary Information

**Supplementary Material 1.**

**Supplementary Material 2.**

## Data Availability

All DNA sequences generated are available from the GenBank database. GenBank accession numbers of all samples included in this study are provided in Table S1. Alignment matrixes and phylogenetic trees are available from Figshare (https://figshare.com/s/f38c8ab9de141d677973).
