# Supplementary material for: Species delimitation of tea plants (Camellia sect. Thea) based on super-barcodes
Source: BMC Plant Biol. 2024 Mar 11;24:181. doi: 10.1186/s12870-024-04882-3 (PMC10926627; doi:10.1186/s12870-024-04882-3)
Supplement: Supplementary file 1 — Supplementary Material 1. [file 12870_2024_4882_MOESM1_ESM.docx]

**Supplementary information**

**Figure S1** Comparisons of maximum likelihood (ML) tree and Bayesian inference (BI) tree based on the plastomes of 167 samples of *Camellia* sect. *Thea*, including two outgroups. A represents the ML tree and B represents BI tree. Bootstrap support value and Bayesian posterior probability are illustrated above the branch.

**Figure S2** Quartet Sampling (QS) scores (QC/QD/QI) shown for the ML tree of *Camellia* sect. *Thea*.

**Table S1** Samples included in the current study with voucher, locality, and GenBank accessions.

**Table S2** Summary of QF scores of QS analysis.





**Figure S1** Comparisons of maximum likelihood (ML) tree and Bayesian inference (BI) tree based on the plastomes of 167 samples of *Camellia* sect. *Thea*, including two outgroups. A represents the ML tree and B represents BI tree. Bootstrap support value and Bayesian posterior probability are illustrated above the branch.

**

**

**Figure S2** Quartet Sampling (QS) scores (QC/QD/QI) shown for the ML tree of *Camellia* sect. *Thea*.

**Table S1** Samples included in the current study with voucher, locality, sequencing technique, and GenBank accessions.

| Voucher | | Species | Locality | Sequence | Reads  number | Length (ex. one IR) (bp) | Gap | Mean coverage | Genbank accession |
| --- | --- | --- | --- | --- | --- | --- | --- | --- | --- |
| YangSX | 2759 | *Camellia arborescens* | Daguan, Yunnan, China | LPCR | 2,011,810 | 130,924 | N | 4920.6 | OQ852472 |
| YangSX | 2909 | *C. arborescens* | Weixin, Yunnan, China | LPCR | 22,671,678 | 130,860 | R | 326.8 | OQ645994 |
| YangSX | 4849_1 | *C. atrothea* | Pingbian, Yunnan, China | LPCR | 1,264,210 | 131,235 | N | 1,055.60 | OR415821 |
| YangSX | 4849_2 | *C. atrothea* | Pingbian, Yunnan, China | LPCR | 1,623,808 | 131,411 | N | 532.4 | OR423139 |
| YangSX | 4849_5 | *C. atrothea* | Pingbian, Yunnan, China | LPCR | 1,555,976 | 131,290 | N | 980.9 | OR423116 |
| YangSX | 5248 | *C. changningensis* | Changning, Yunnan, China | LPCR | 2,409,382 | 130,812 | N | 2225.839 | OR415812 |
| YangSX | 4735 | *C. costata* | Zhaoping, Guangxi, China | LPCR | 1,131,366 | 133,043 | N | 942.5 | OQ852476 |
| YangSX | 4760 | *C. costata* | Zhaoping, Guangxi, China | LPCR | 615,172 | 130,932 | N | 662.3 | OQ852477 |
| YangSX | 4761 | *C. costata* | Zhaoping, Guangxi, China | LPCR | 512,672 | 131,395 | N | 646.2 | OR423128 |
| YangSX | 4736_1 | *C. costata* | Zhaoping, Guangxi, China | Geskim | 19,058,064 | 131,126 | R | 511.6 | OQ646004 |
| YangSX | 5527_1 | *C. costata* | Zhaoping, Guangxi, China | Geskim | 30,000,000 | 130,863 | R | NA | OQ646038 |
| YangSX | 5527_2 | *C. costata* | Zhaoping, Guangxi, China | LPCR | 9,902,500 | 130,863 | R | 100.9 | OQ646039 |
| YangSX | 5527_3 | *C. costata* | Zhaoping, Guangxi, China | LPCR | 12,113,972 | 130,863 | R | 370 | OQ646040 |
| YangSX | 5527_4 | *C. costata* | Zhaoping, Guangxi, China | LPCR | 17,677,464 | 130,863 | R | 224.9 | OQ646041 |
| YangSX | 4852 | *C. crispula* | Pingbian, Yunnan, China | Geskim | 30,000,000 | 130,939 | R | NA | OQ646010 |
| YangSX | 4875 | *C. crispula* | Jinping, Yunnan, China | Geskim | 30,000,000 | 130,568 | R | NA | OQ646014 |
| YangSX | 4876 | *C. crispula* | Jinping, Yunnan, China | LPCR | 2,038,632 | 131,212 | N | 852.6 | OR415784 |
| YangSX | 4877 | *C. crispula* | Jinping, Yunnan, China | LPCR | 2,269,934 | 131,199 | N | 931.6 | OR415785 |
| YangSX | 4878 | *C. crispula* | Jinping, Yunnan, China | LPCR | 1,985,026 | 131,183 | N | 854.2 | OR415786 |
| YangSX | 4853_1 | *C. crispula* | Pingbian, Yunnan, China | LPCR | 1,201,906 | 131,106 | N | 1,103.60 | OR415824 |
| YangSX | 4853_3 | *C. crispula* | Pingbian, Yunnan, China | LPCR | 1,270,210 | 131,060 | N | 1,829.20 | OR415825 |
| YangSX | 2635 | *C. crassicolumna* var. *shangbaensis* | Zhenyuan, Yunnan, China | Geskim | 30,000,000 | 131,000 | R | 385.3 | OQ645990 |
| YangSX | 2659 | *C. crassicolumna* var. *shangbaensis* | Zhenyuan, Yunnan, China | LPCR | 21,806,112 | 131,000 | R | 229.6 | OQ645991 |
| YangSX | 2664 | *C. crassicolumna* var. *shangbaensis* | Zhenyuan, Yunnan, China | LPCR | 26,688,864 | 131,000 | R | 306.7 | OQ645992 |
| YangSX | 3150_A | *C. danzaiensis* | Danzhai, Guizhou, China | Geskim | 30,000,000 | 130,338 | R | NA | OQ645995 |
| YangSX | 5082_1 | *C. danzaiensis* | Danzhai, Guizhou, China | Geskim | 30,000,000 | 130,347 | R | 514 | OQ646027 |
| YangSX | 5082_2A | *C. danzaiensis* | Danzhai, Guizhou, China | LPCR | 1,929,142 | 131,375 | N | 2,437.20 | OR423135 |
| YangSX | 5241 | *C. dehungensis* | Shidian, Yunnan, China | Geskim | 30,000,000 | 131,011 | R | 463 | OR423135 |
| YangSX | 4803_A | *C. dishiensis* | Guangnan, Yunnan, China | LPCR | 829,748 | 131,514 | N | 1,249.00 | OR415773 |
| YangSX | 4803_C | *C. dishiensis* | Guangnan, Yunnan, China | LPCR | 1,299,806 | 131,194 | N | 1,419.20 | OR415774 |
| YangSX | 4768_1 | *C. fangchengensis* | Guilin (introduced from Fangcheng), Guangxi, China | Geskim | 24,941,904 | 130,768 | R | NA | OR415773 |
| YangSX | 4768_3 | *C. fangchengensis* | Guilin (introduced from Fangcheng), Guangxi, China | LPCR | 1,198,012 | 130,905 | N | 978.5 | OR415815 |
| YangSX | 4768_4 | *C. fangchengensis* | Guilin (introduced from Fangcheng), Guangxi, China | Geskim | 30,000,000 | 130,768 | R | NA | OQ646052 |
| YangSX | 5035_15 | *C. formosensis* | Kaohsiung, Taiwan, China | LPCR | 1,638,028 | 131,481 | N | 2,034.50 | OR423142 |
| YangSX | 5036_15 | *C. formosensis* | Kaohsiung, Taiwan, China | Geskim | 30,000,000 | 130,973 | R | 71.7 | OQ646022 |
| YangSX | 5036_18 | *C. formosensis* | Kaohsiung, Taiwan, China | LPCR | 1,518,408 | 131,400 | N | 2,359.80 | OR415797 |
| YangSX | 5036_20 | *C. formosensis* | Kaohsiung, Taiwan, China | Geskim | 30,000,000 | 130,973 | R | 105.2 | OQ646023 |
| YangSX | 5036_6 | *C. formosensis* | Kaohsiung, Taiwan, China | Geskim | 30,000,000 | 130,973 | R | 206.3 | OQ646021 |
| YangSX | 5039_13 | *C. formosensis* | Pingtung, Taiwan, China | LPCR | 1,295,784 | 131,153 | N | 1,117.30 | OR415798 |
| YangSX | 5039_17 | *C. formosensis* | Pingtung, Taiwan, China | LPCR | 1,794,550 | 131,277 | N | 1,015.80 | OR415835 |
| YangSX | 4850_2 | *C. glaberrima* | Pingbian, Yunnan, China | Geskim | 30,000,000 | 130,623 | R | 419.6 | OQ646053 |
| YangSX | 4850_3 | *C. glaberrima* | Pingbian, Yunnan, China | LPCR | 1,502,976 | 131,430 | N | 1,501.70 | OR415822 |
| YangSX | 4850_4 | *C. glaberrima* | Pingbian, Yunnan, China | LPCR | 1,592,412 | 131,166 | N | 2,236.40 | OR415823 |
| YangSX | 4600 | *C. grandibracteata* | Yunxian, Yunnan, China | Geskim | 30,000,000 | 131,058 | R | NA | OQ646002 |
| YangSX | 4602 | *C. grandibracteata* | Yunxian, Yunnan, China | LPCR | 5,033,184 | 131,265 | N | 516 | OR423124 |
| YangSX | 4605 | *C. grandibracteata* | Yunxian, Yunnan, China | Geskim | 30,000,000 | 131,058 | R | NA | OQ646003 |
| YangSX | 4654 | *C. gymnogyna* | Tianlin, Guangxi, China | LPCR | 386,556 | 130,833 | N | 440.2 | OR423127 |
| YangSX | 4656 | *C. gymnogyna* | Tianlin, Guangxi, China | LPCR | 251,240 | 130,839 | N | 257.2 | OQ852475 |
| YangSX | 4329_3 | *C. gymnogyna* | Tianlin, Guangxi, China | LPCR | 1,193,278 | 130,920 | N | 1,200.40 | OR423122 |
| YangSX | 4869 | *C. haaniensis* | Jinping, Yunnan, China | LPCR | 1,326,490 | 131,964 | N | 1,460.60 | OR415781 |
| YangSX | 4870 | *C. haaniensis* | Jinping, Yunnan, China | LPCR | 1,488,174 | 131,165 | N | 2,179.90 | OR423130 |
| YangSX | 4871 | *C. haaniensis* | Jinping, Yunnan, China | LPCR | 935,782 | 131,735 | N | 959.3 | OR415782 |
| YangSX | 4872 | *C. haaniensis* | Jinping, Yunnan, China | LPCR | 1,349,402 | 131,776 | N | 1,619.90 | OR415783 |
| YangSX | 4781 | *C. kwangnanica* | Guangnan, Yunnan, China | Geskim | 30,000,000 | 130,959 | R | NA | OQ646005 |
| YangSX | 4792 | *C. kwangnanica* | Guangnan, Yunnan, China | LPCR | 1,296,770 | 131,640 | N | 1,968.30 | OQ852478 |
| YangSX | 4808 | *C. kwangnanica* | Funing, Yunnan, China | Geskim | 30,000,000 | 130,977 | R | 336 | OQ646006 |
| YangSX | 4809_A | *C. kwangnanica* | Funing, Yunnan, China | LPCR | 1,186,694 | 131,182 | N | 1,691.30 | OR415775 |
| YangSX | 4810_A | *C. kwangnanica* | Funing, Yunnan, China | LPCR | 1,006,402 | 131,603 | N | 1,322.50 | OR415776 |
| YangSX | 4788 | *C. kwangsiensis* | Guangnan, Yunnan, China | LPCR | 1,384,660 | 131,896 | N | 1,876.90 | OR423129 |
| YangSX | 4793 | *C. kwangsiensis* | Guangnan, Yunnan, China | LPCR | 1,141,984 | 131,179 | N | 1,414.40 | OR415772 |
| YangSX | 4822 | *C. kwangsiensis* | Malipo, Yunnan, China | LPCR | 1,832,458 | 131,186 | N | 2,676.10 | OR415777 |
| YangSX | 4823 | *C. kwangsiensis* | Malipo, Yunnan, China | LPCR | 1,926,856 | 131,044 | N | 1,885.80 | OR415778 |
| YangSX | 4824 | *C. kwangsiensis* | Malipo, Yunnan, China | Geskim | 17,038,954 | 130,936 | R | 415.9 | OQ646007 |
| YangSX | 4825_A | *C. kwangsiensis* | Malipo, Yunnan, China | LPCR | 1,265,442 | 131,625 | N | 1,777.60 | OR415779 |
| YangSX | 5543 | *C. kwangtungensis* | Qingyuan, Guangdong, China | Geskim | 30,000,000 | 130,636 | R | 167.5 | OQ646042 |
| YangSX | 5546 | *C. kwangtungensis* | Qingyuan, Guangdong, China | Geskim | 30,000,000 | 131,002 | R | 83.3 | OQ646043 |
| YangSX | 5073 | *C. leptophylla* | Longzhou, Guangxi, China | Geskim | 30,000,000 | 131,002 | R | NA | OQ646025 |
| YangSX | 5074 | *C. leptophylla* | Longzhou, Guangxi, China | LPCR | 1,728,868 | 131,542 | N | 2,759.30 | OR415805 |
| YangSX | 5075 | *C. leptophylla* | Longzhou, Guangxi, China | Geskim | 30,000,000 | 131,002 | R | NA | OQ646026 |
| YangSX | 5076 | *C. leptophylla* | Longzhou, Guangxi, China | LPCR | 1,966,440 | 131,443 | N | 2,271.50 | OR423133 |
| YangSX | 6660 | *C. leptophylla* | Longzhou, Guangxi, China | Geskim | 30,000,000 | 130,936 | R | NA | OQ646049 |
| YangSX | 6661_1 | *C. leptophylla* | Longzhou, Guangxi, China | Geskim | 30,000,000 | 130,936 | R | NA | OQ646050 |
| YangSX | 5252 | *C. longlingensis* | Longling, Yunnan, China | LPCR | 1,442,088 | 130,612 | N | 851 | OR423137 |
| YangSX | 4838 | *C. makuanica* | Maguan, Yunnan, China | Geskim | 30,000,000 | 130,314 | R | 117.5 | OQ646008 |
| YangSX | 4844 | *C. makuanica* | Maguan, Yunnan, China | Geskim | 30,000,000 | 130,329 | R | 436.7 | OQ646009 |
| YangSX | 4840_1 | *C. makuanica* | Maguan, Yunnan, China | LPCR | 1,221,492 | 131,219 | N | 991.5 | OR415818 |
| YangSX | 4843_1 | *C. makuanica* | Maguan, Yunnan, China | LPCR | 3,617,616 | 131,079 | N | 946.9 | OR415819 |
| YangSX | 4845_1 | *C. makuanica* | Maguan, Yunnan, China | LPCR | 1,111,210 | 131,086 | N | 1,047.70 | OR415820 |
| YangSX | 4901 | *C. multiplex* | Lvechun, Yunnan, China | Geskim | 30,000,000 | 130,929 | R | NA | OQ646018 |
| YangSX | 4902 | *C. multiplex* | Lvechun, Yunnan, China | LPCR | 1,579,580 | 131,268 | N | 2,059.00 | OR415791 |
| YangSX | 4904 | *C. multiplex* | Lvechun, Yunnan, China | LPCR | 1,313,884 | 131,035 | N | 1,369.40 | OR415792 |
| YangSX | 4913 | *C. multiplex* | Jianshui, Yunnan, China | Geskim | 30,000,000 | 130,969 | R | NA | OQ646020 |
| YangSX | 4837_1 | *C. multiplex* | Wenshan, Yunnan, China | LPCR | 1,622,780 | 131,302 | N | 1,866.10 | OR415817 |
| YangSX | 4906_1 | *C. multiplex* | Lvechun, Yunnan, China | Geskim | 30,000,000 | 130,929 | R | NA | OQ646019 |
| YangSX | 4914 | *C. multiplex* | Jianshui, Yunnan, China | LPCR | 874,176 | 131,173 | N | 1,192.30 | OR415793 |
| YangSX | 6792_A | *C. nanchuanica* | Nanchuan, chongqin, China | LPCR | 1,593,854 | 131,303 | N | 1,650.30 | OR415813 |
| YangSX | 6792_B | *C. nanchuanica* | Nanchuan, chongqin, China | LPCR | 1,276,174 | 131,507 | N | 1,527.20 | OR423138 |
| YangSX | 3871_1 | *C. parvisepala* | Lingyun, Guangxi, China | LPCR | 26,069,174 | 131,008 | R | 184.4 | OQ645997 |
| YangSX | 3872_1 | *C. parvisepala* | Lingyun, Guangxi, China | LPCR | 26,709,038 | 131,008 | R | 188.1 | OQ645998 |
| YangSX | 6491 | *C. parvisepaloides* | Mangshi, Yunnan, China | Geskim | 30,000,000 | 131,009 | R | 504.9 | OQ646048 |
| YangSX | 4591 | *C. pentastyla* | Fengqing, Yunnan, China | LPCR | 1,775,030 | 131,071 | N | 3,088.80 | OR423123 |
| YangSX | 4898_1 | *C. polyneura* | Lvechun, Yunnan, China | Geskim | 30,000,000 | 130,939 | R | 413.2 | OQ646015 |
| YangSX | 4898_2 | *C. polyneura* | Lvechun, Yunnan, China | Geskim | 30,000,000 | 130,939 | R | 344.6 | OQ646016 |
| YangSX | 4898_3 | *C. polyneura* | Lvechun, Yunnan, China | Geskim | 30,000,000 | 130,939 | R | 402.1 | OQ646017 |
| YangSX | 4898_4 | *C. polyneura* | Lvechun, Yunnan, China | LPCR | 1,251,696 | 131,090 | N | 556.4 | OR415787 |
| YangSX | 5107_1 | *C. ptilophylla* | Longmen, Guangdong, China | LPCR | 1,452,182 | 131,199 | N | 1,765.90 | OR415811 |
| YangSX | 5107_2A | *C. ptilophylla* | Longmen, Guangdong, China | LPCR | 1,808,556 | 131,442 | N | 1,861.10 | OR415833 |
| YangSX | 5107_3A | *C. ptilophylla* | Longmen, Guangdong, China | LPCR | 1,471,122 | 131,155 | N | 1,618.70 | OR415834 |
| YangSX | 4455 | *C. pubescens* | Rucheng, Hunan, China | Geskim | 30,000,000 | 130,445 | R | NA | OQ646000 |
| YangSX | 4457 | *C. pubescens* | Rucheng, Hunan, China | LPCR | 4,609,274 | 131,066 | N | 1,202.60 | OQ852473 |
| YangSX | 4458 | *C. pubescens* | Rucheng, Hunan, China | Geskim | 30,000,000 | 130,445 | R | NA | OQ646001 |
| YangSX | 4323_3 | *C. quinquelocularis* | Longlin, Guangxi, China | LPCR | 1,702,368 | 131,355 | N | 1,921.90 | OR423119 |
| YangSX | 4323_7 | *C. quinquelocularis* | Longlin, Guangxi, China | LPCR | 1,626,468 | 131,230 | N | 1,108.80 | OR423120 |
| YangSX | 4323_9 | *C. quinquelocularis* | Longlin, Guangxi, China | LPCR | 1,708,754 | 131,323 | N | 1,067.90 | OR423121 |
| YangSX | 5048 | *C. remotiserrata* | Yibin, Sichuan, China | LPCR | 1,844,506 | 131,182 | N | 2,169.40 | OR415799 |
| YangSX | 5049 | *C. remotiserrata* | Yibin, Sichuan, China | LPCR | 1,931,422 | 131,076 | N | 2,395.70 | OR423132 |
| YangSX | 5072 | *C. remotiserrata* | Yibin, Sichuan, China | Geskim | 30,000,000 | 130,787 | R | 499.6 | OQ646024 |
| YangSX | 2906_1 | *C. remotiserrata* | Weixin, Yunnan, China | Geskim | 30,000,000 | 130,920 | R | 209.7 | OQ645993 |
| YangSX | 5071_5 | *C. remotiserrata* | Yibin, Sichuan, China | LPCR | 1,311,428 | 131,036 | N | 1,502.80 | OR415802 |
| YangSX | 5071_6 | *C. remotiserrata* | Yibin, Sichuan, China | LPCR | 1,074,206 | 130,939 | N | 1,421.70 | OR415803 |
| YangSX | 5071_7 | *C. remotiserrata* | Yibin, Sichuan, China | LPCR | 1,367,276 | 131,060 | N | 1,892.40 | OR415804 |
| YangSX | 5298_1 | *C. remotiserrata* | Chishui, Guizhou, China | Geskim | 17,317,132 | 130,860 | R | 422.7 | OQ646031 |
| YangSX | 4770_2 | *C. sinensis* var. *sinensis* | Hangzhou, Zhejiang, China | LPCR | 866,938 | 131,102 | N | 511.5 | OR415816 |
| YangSX | 4936_4 | *C. sinensis* var. *sinensis* | Meitan, Guizhou, China | LPCR | 1,978,666 | 131,185 | N | 551.9 | OR415828 |
| YangSX | 4938_2 | *C. sinensis* var. *sinensis* | Shiqian, Guizhou, China | LPCR | 1,167,058 | 131,166 | N | 670.1 | OR415794 |
| YangSX | 4939_1 | *C. sinensis* var. *sinensis* | Shiqian, Guizhou, China | LPCR | 1,416,606 | 131,247 | N | 650.3 | OR415795 |
| YangSX | 4941_6 | *C. sinensis* var. *sinensis* | Duyun, Guizhou, China | LPCR | 1,111,482 | 131,227 | N | 588.9 | OR415796 |
| YangSX | 4962_4 | *C. sinensis var. sinensis* | Longli, Guizhou, China | LPCR | 1,145,856 | 131,171 | N | 586.1 | OR423131 |
| YangSX | 4613 | *C. sinensis* var. *assamica* | Shuangjiang, Yunnan, China | LPCR | 4,720,292 | 131,375 | N | 1,065.90 | OR423125 |
| YangSX | 4618 | *C. sinensis* var. *assamica* | Shuangjiang, Yunnan, China | LPCR | 5,037,116 | 131,240 | N | 1,082.70 | OR423126 |
| YangSX | 4862 | *C. sinensis* var. *assamica* | Jinping, Yunnan, China | LPCR | 1,652,518 | 131,214 | N | 576.4 | OR415780 |
| YangSX | 5335 | *C. sinensis* var. *assamica* | Laos | LPCR | 14,463,660 | 131,022 | R | 461.8 | OQ646032 |
| YangSX | 5336 | *C. sinensis* var. *assamica* | Laos | LPCR | 14,506,698 | 131,028 | R | 413.2 | OQ646033 |
| YangSX | 5337 | *C. sinensis* var. *assamica* | Laos | LPCR | 14,499,046 | 131,028 | R | 170.6 | OQ646034 |
| YangSX | 4867_5 | *C. sinensis* var. *assamica* | Jinping, Yunnan, China | LPCR | 1,778,440 | 131,150 | N | 1,395.50 | OR423140 |
| YangSX | 4899_1 | *C. sinensis* var. *assamica* | Jinping, Yunnan, China | LPCR | 1,071,322 | 131,205 | N | 586.1 | OR415788 |
| YangSX | 4899_2 | *C. sinensis* var. *assamica* | Jinping, Yunnan, China | LPCR | 1,436,660 | 131,191 | N | 560.5 | OR415789 |
| YangSX | 4899_3 | *C. sinensis* var. *assamica* | Jinping, Yunnan, China | LPCR | 1,502,732 | 131,135 | N | 551.7 | OR415790 |
| YangSX | 4858 | *C. sinensis* var. *kucha* | Jinping, Yunnan, China | Geskim | 27,163,032 | 130,567 | R | 382.5 | OQ646011 |
| YangSX | 4859 | *C. sinensis* var. *kucha* | Jinping, Yunnan, China | Geskim | 30,000,000 | 131,006 | R | 427 | OQ646012 |
| YangSX | 4857_1 | *C. sinensis* var. *kucha* | Jinping, Yunnan, China | LPCR | 1,125,272 | 131,172 | N | 972.4 | OR415826 |
| YangSX | 4860_2 | *C. sinensis* var. *kucha* | Jinping, Yunnan, China | LPCR | 1,153,446 | 131,154 | N | 1,468.20 | OR415827 |
| YangSX | 4860_1 | *C. sinensis* var. *kucha* | Jinping, Yunnan, China | Geskim | 30,000,000 | 131,006 | N | 294.8 | OQ646013 |
| YangSX | 4629 | *C. sinensis* var. *pubilimba* | Fusui, Guangxi, China | LPCR | 486,852 | 131,501 | N | 591.9 | OQ852474 |
| YangSX | 4707_2 | *C. sinensis* var. *pubilimba* | Longsheng, Guangxi, China | LPCR | 1,050,876 | 131,169 | N | 558.6 | OR415814 |
| YangSX | 5062_1 | *C. sinensis* var. *pubilimba* | Chongzhou, Sichuan, China | LPCR | 2,356,912 | 131,002 | N | 2,786.30 | OR415800 |
| YangSX | 5062_3 | *C. sinensis* var. *pubilimba* | Chongzhou, Sichuan, China | LPCR | 1,281,126 | 130,982 | N | 1,716.40 | OR415829 |
| YangSX | 5062_5 | *C. sinensis* var. *pubilimba* | Chongzhou, Sichuan, China | LPCR | 1,356,150 | 130,990 | N | 1,848.70 | OR415801 |
| YangSX | 5078_1 | *C. sinensis* var. *pubilimba* | Longzhou, Guangxi, China | LPCR | 1,531,148 | 131,210 | R | 1,992.00 | OR423134 |
| YangSX | 5078_2 | *C. sinensis* var. *pubilimba* | Longzhou, Guangxi, China | LPCR | 1,732,556 | 131,111 | N | 2,344.40 | OR415806 |
| YangSX | 5504_1 | *C. sinensis* var. *pubilimba* | Luocheng, Guangxi, China | LPCR | 21,595,748 | 131,008 | R | 371.7 | OQ646035 |
| YangSX | 5504_2 | *C. sinensis* var. *pubilimba* | Luocheng, Guangxi, China | LPCR | 21,915,252 | 131,177 | R | 370.6 | OQ646036 |
| YangSX | 5504_3 | *C. sinensis* var. *pubilimba* | Luocheng, Guangxi, China | LPCR | 25,270,596 | 130,966 | R | 393.1 | OQ646037 |
| YangSX | 4149 | *C. sinensis* var. *pubilimba* | Jinxiu, Guangxi, China | LPCR | 692,930 | 130,851 | N | 773.6 | OR423117 |
| YangSX | 4199 | *C. sinensis* var. *pubilimba* | Zhaoping, Guangxi, China | LPCR | 24,575,780 | 130,854 | R | 358.5 | OQ645999 |
| YangSX | 5102 | *C. tachangensis* | Qinglong, Guizhou, China | LPCR | 1,421,502 | 131,073 | N | 1,927.80 | OR415807 |
| YangSX | 4319_4 | *C. tachangensis* | Xingyi, Guizhou, China | LPCR | 1,702,088 | 131,009 | N | 3,153.90 | OR423118 |
| DengCY | D12050 | *C. tachangensis* | Xingyi, Guizhou, China | Geskim | 11,077,118 | 131,004 | R | 737.7 | OQ646054 |
| DengCY | D12051 | *C. tachangensis* | Xingyi, Guizhou, China | LPCR | 2,093,012 | 131,163 | N | 1,646.80 | OR415837 |
| DengCY | D12052 | *C. tachangensis* | Xingyi, Guizhou, China | Geskim | 26,234,454 | 130,974 | R | NA | OQ646055 |
| YangSX | 3157 | *C. taliensis* | Zhenyuan, Yunnan, China | Geskim | 30,000,000 | 130,951 | R | 298.7 | OQ645996 |
| YangSX | 5566 | *C. taliensis* | Baoshan, Yunnan, China | Geskim | 30,000,000 | 130,622 | R | NA | OQ646044 |
| YangSX | 5579_1 | *C. taliensis* | Tengchong, Yunnan, China | Geskim | 30,000,000 | 130,971 | R | 418.4 | OQ646045 |
| YangSX | 5585_1 | *C. taliensis* | Lincang, Yunnan, China | Geskim | 30,000,000 | 130,947 | R | 516.5 | OQ646046 |
| YangSX | 5104_1 | *C. tetracarpa* | Puan, Guizhou, China | LPCR | 983,664 | 131,096 | N | 1,249.50 | OR415808 |
| YangSX | 5104_2 | *C. tetracarpa* | Puan, Guizhou, China | LPCR | 1,829,350 | 130,929 | N | 2,193.20 | OR423136 |
| YangSX | 5104_5 | *C. tetracarpa* | Puan, Guizhou, China | LPCR | 1,253,674 | 130,950 | N | 1,327.30 | OR415809 |
| YangSX | 5105_24A | *C. tetracarpa* | Puan, Guizhou, China | LPCR | 1,273,102 | 131,173 | N | 1,176.10 | OR415810 |
| YangSX | 5105_25A | *C. tetracarpa* | Puan, Guizhou, China | LPCR | 2,821,588 | 131,114 | N | 2,166.00 | OR415836 |
| YangSX | 5092_1 | *C. yungkiangensis* | Rongjiang, Guizhou, China | Geskim | 30,000,000 | 130,497 | R | 502.1 | OQ646028 |
| YangSX | 5092_2A | *C. yungkiangensis* | Rongjiang, Guizhou, China | LPCR | 2,093,798 | 130,538 | N | 2785.6 | OR415830 |
| YangSX | 5092_3A | *C. yungkiangensis* | Leishan, Guizhou, China | LPCR | 1,573,514 | 130,492 | N | 2213.8 | OR415831 |
| YangSX | 5095_1 | *C. yungkiangensis* | Leishan, Guizhou, China | Geskim | 29,278,880 | 130,465 | R | 528.8 | OQ646029 |
| YangSX | 5095_2A | *C. yungkiangensis* | Leishan, Guizhou, China | LPCR | 2,417,430 | 130,862 | N | 2963.2 | OR423141 |
| YangSX | 5095_4A | *C. yungkiangensis* | Leishan, Guizhou, China | LPCR | 1,796,428 | 130,460 | N | 2552.5 | OR415832 |

Note: “LPCR” means the data was produced by Long-range PCR, and “Geskim” means the data was produced by genome skimming data. “Reads number” means the amount of reads used for assembling plastomes. “R” indicates the plastome was complete. “N” indictes the plastome was assembled to nearly complete (with gaps).

**Table S2** Summary of QF scores of QS analysis.

| Sample ID | Species | QF |
| --- | --- | --- |
| 2759 | *Camellia arborescens* | 0.705686731 |
| 2909 | *C. arborescens* | 0.919145371 |
| 4849_1 | *C. atrothea* | 0.850756776 |
| 4849_2 | *C. atrothea* | 0.824611033 |
| 4849_5 | *C. atrothea* | 0.892607241 |
| 5248 | *C. changningensis* | 0.733265375 |
| 4735 | *C. costata* | 0.673675698 |
| 4760 | *C. costata* | 0.784913354 |
| 4761 | *C. costata* | 0.714825703 |
| 4736_1 | *C. costata* | 0.720379147 |
| 5527_1 | *C. costata* | 0.661454261 |
| 5527_2 | *C. costata* | 0.714223952 |
| 5527_3 | *C. costata* | 0.708103521 |
| 5527_4 | *C. costata* | 0.767364573 |
| 4852 | *C. crispula* | 0.816290131 |
| 4875 | *C. crispula* | 0.875402447 |
| 4876 | *C. crispula* | 0.648573417 |
| 4877 | *C. crispula* | 0.648275862 |
| 4878 | *C. crispula* | 0.647474391 |
| 4853_1 | *C. crispula* | 0.812894184 |
| 4853_3 | *C. crispula* | 0.835326273 |
| 2635 | *C. crassicolumna* var. *shangbaensis* | 0.774311183 |
| 2659 | *C. crassicolumna* var. *shangbaensis* | 0.774284563 |
| 2664 | *C. crassicolumna* var. *shangbaensis* | 0.769587195 |
| 3150_A | *C. danzaiensis* | 0.784670232 |
| 5082_1 | *C. danzaiensis* | 0.720624486 |
| 5082_2A | *C. danzaiensis* | 0.684210526 |
| 4803_A | *C. dishiensis* | 0.854395604 |
| 4803_C | *C. dishiensis* | 0.850320741 |
| 4768_1 | *C. fangchengensis* | 0.687545257 |
| 4768_3 | *C. fangchengensis* | 0.705516989 |
| 4768_4 | *C. fangchengensis* | 0.729354839 |
| 5035_15 | *C. formosensis* | 0.90389765 |
| 5036_15 | *C. formosensis* | 0.81969112 |
| 5036_18 | *C. formosensis* | 0.870263716 |
| 5036_20 | *C. formosensis* | 0.813657407 |
| 5036_6 | *C. formosensis* | 0.806463196 |
| 5039_13 | *C. formosensis* | 0.912566594 |
| 5039_17 | *C. formosensis* | 0.917271866 |
| 4850_2 | *C. glaberrima* | 0.816917729 |
| 4850_3 | *C. glaberrima* | 0.85286285 |
| 4850_4 | *C. glaberrima* | 0.823708207 |
| 4600 | *C. grandibracteata* | 0.876588022 |
| 4602 | *C. grandibracteata* | 0.889669927 |
| 4605 | *C. grandibracteata* | 0.874362709 |
| 4654 | *C. gymnogyna* | 0.811233558 |
| 4656 | *C. gymnogyna* | 0.809661139 |
| 4329_3 | *C. gymnogyna* | 0.75764355 |
| 4869 | *C. haaniensis* | 0.644849785 |
| 4870 | *C. haaniensis* | 0.781888133 |
| 4871 | *C. haaniensis* | 0.65408805 |
| 4872 | *C. haaniensis* | 0.572844698 |
| 4781 | *C. kwangnanica* | 0.868039443 |
| 4792 | *C. kwangnanica* | 0.774742817 |
| 4808 | *C. kwangnanica* | 0.87041481 |
| 4809_A | *C. kwangnanica* | 0.868432114 |
| 4810_A | *C. kwangnanica* | 0.854537744 |
| 4788 | *C. kwangsiensis* | 0.721566976 |
| 4793 | *C. kwangsiensis* | 0.724516696 |
| 4822 | *C. kwangsiensis* | 0.783089688 |
| 4823 | *C. kwangsiensis* | 0.726838966 |
| 4824 | *C. kwangsiensis* | 0.74746292 |
| 4825_A | *C. kwangsiensis* | 0.779225908 |
| 5543 | *C. kwangtungensis* | 0.89586071 |
| 5546 | *C. kwangtungensis* | 0.875279821 |
| 5073 | *C. leptophylla* | 0.727108216 |
| 5074 | *C. leptophylla* | 0.818548387 |
| 5075 | *C. leptophylla* | 0.712875047 |
| 5076 | *C. leptophylla* | 0.857457752 |
| 6660 | *C. leptophylla* | 0.828690229 |
| 6661_1 | *C. leptophylla* | 0.825692042 |
| 5252 | *C. longlingensis* | 0.734892788 |
| 4838 | *C. makuanica* | 0.853928299 |
| 4844 | *C. makuanica* | 0.727431313 |
| 4840_1 | *C. makuanica* | 0.873128743 |
| 4843_1 | *C. makuanica* | 0.813922356 |
| 4845_1 | *C. makuanica* | 0.782205029 |
| 4901 | *C. multiplex* | 0.622828784 |
| 4902 | *C. multiplex* | 0.583733974 |
| 4904 | *C. multiplex* | 0.676771005 |
| 4913 | *C. multiplex* | 0.739010038 |
| 4837_1 | *C. multiplex* | 0.863557257 |
| 4906_1 | *C. multiplex* | 0.645399306 |
| 4914 | *C. multiplex* | 0.765768958 |
| 6792_A | *C. nanchuanica* | 0.913375796 |
| 6792_B | *C. nanchuanica* | 0.923432899 |
| 3871_1 | *C. parvisepala* | 0.682725832 |
| 3872_1 | *C. parvisepala* | 0.682263674 |
| 6491 | *C. parvisepaloides* | 0.749643976 |
| 4591 | *C. pentastyla* | 0.858510967 |
| 4898_1 | *C. polyneura* | 0.748189178 |
| 4898_2 | *C. polyneura* | 0.742990654 |
| 4898_3 | *C. polyneura* | 0.730288837 |
| 4898_4 | *C. polyneura* | 0.755733527 |
| 5107_1 | *C. ptilophylla* | 0.724481328 |
| 5107_2A | *C. ptilophylla* | 0.621557293 |
| 5107_3A | *C. ptilophylla* | 0.725612287 |
| 4455 | *C. pubescens* | 0.667292527 |
| 4457 | *C. pubescens* | 0.710466989 |
| 4458 | *C. pubescens* | 0.666912851 |
| 4323_3 | *C. quinquelocularis* | 0.680309231 |
| 4323_7 | *C. quinquelocularis* | 0.692801467 |
| 4323_9 | *C. quinquelocularis* | 0.75764355 |
| 5048 | *C. remotiserrata* | 0.802531646 |
| 5049 | *C. remotiserrata* | 0.806381436 |
| 5072 | *C. remotiserrata* | 0.744143596 |
| 2906_1 | *C. remotiserrata* | 0.927297206 |
| 5071_5 | *C. remotiserrata* | 0.82292432 |
| 5071_6 | *C. remotiserrata* | 0.842154132 |
| 5071_7 | *C. remotiserrata* | 0.827387387 |
| 5298_1 | *C. remotiserrata* | 0.913735343 |
| 4770_2 | *C. sinensis* var. *sinensis* | 0.612668744 |
| 4936_4 | *C. sinensis* var. *sinensis* | 0.672550213 |
| 4938_2 | *C. sinensis* var. *sinensis* | 0.610198494 |
| 4939_1 | *C. sinensis* var. *sinensis* | 0.462582886 |
| 4941_6 | *C. sinensis* var. *sinensis* | 0.600702988 |
| 4962_4 | *C. sinensis* var. *sinensis* | 0.628434066 |
| 4613 | *C. sinensis* var. *assamica* | 0.79801207 |
| 4618 | *C. sinensis* var. *assamica* | 0.768265208 |
| 4862 | *C. sinensis* var. *assamica* | 0.832428982 |
| 5335 | *C. sinensis* var. *assamica* | 0.933375837 |
| 5336 | *C. sinensis* var. *assamica* | 0.941505912 |
| 5337 | *C. sinensis* var. *assamica* | 0.935860979 |
| 4867_5 | *C. sinensis* var. *assamica* | 0.826351135 |
| 4899_1 | *C. sinensis* var. *assamica* | 0.837517115 |
| 4899_2 | *C. sinensis* var. *assamica* | 0.867732558 |
| 4899_3 | *C. sinensis* var. *assamica* | 0.84315463 |
| 5241 | *C. sinensis* var. *dehungensis* | 0.805246137 |
| 4858 | *C. sinensis* var. *kucha* | 0.892592593 |
| 4859 | *C. sinensis* var. *kucha* | 0.724169742 |
| 4857_1 | *C. sinensis* var. *kucha* | 0.706889353 |
| 4860_2 | *C. sinensis var. kucha* | 0.753623188 |
| 4860_1 | *C. sinensis var. kucha* | 0.72858473 |
| 4629 | *C. sinensis var. pubilimba* | 0.648427261 |
| 4707_2 | *C. sinensis var. pubilimba* | 0.663736264 |
| 5062_1 | *C. sinensis var. pubilimba* | 0.8776267 |
| 5062_3 | *C. sinensis var. pubilimba* | 0.884077736 |
| 5062_5 | *C. sinensis var. pubilimba* | 0.867019113 |
| 5078_1 | *C. sinensis var. pubilimba* | 0.665353038 |
| 5078_2 | *C. sinensis var. pubilimba* | 0.664912858 |
| 5504_1 | *C. sinensis var. pubilimba* | 0.668592058 |
| 5504_2 | *C. sinensis var. pubilimba* | 0.837652201 |
| 5504_3 | *C. sinensis var. pubilimba* | 0.837390762 |
| 4149 | *C. sinensis var. pubilimba* | 0.703140175 |
| 4199 | *C. sinensis var. pubilimba* | 0.743465634 |
| 5102 | *C. tachangensis* | 0.768101761 |
| 4319_4 | *C. tachangensis* | 0.693258427 |
| D12050 | *C. tachangensis* | 0.867375665 |
| D12051 | *C. tachangensis* | 0.89056 |
| D12052 | *C. tachangensis* | 0.680422582 |
| 3157 | *C. taliensis* | 0.84119876 |
| 5566 | *C. taliensis* | 0.72688588 |
| 5579_1 | *C. taliensis* | 0.847673678 |
| 5585_1 | *C. taliensis* | 0.875240539 |
| 5104_1 | *C. tetracarpa* | 0.723076923 |
| 5104_2 | *C. tetracarpa* | 0.724179343 |
| 5104_5 | *C. tetracarpa* | 0.709954937 |
| 5105_24A | *C. tetracarpa* | 0.696675383 |
| 5105_25A | *C. tetracarpa* | 0.699013158 |
| 5092_1 | *C. yungkiangensis* | 0.84013901 |
| 5092_2A | *C. yungkiangensis* | 0.811962874 |
| 5092_3A | *C. yungkiangensis* | 0.781867757 |
| 5095_1 | *C. yungkiangensis* | 0.792599502 |
| 5095_2A | *C. yungkiangensis* | 0.750649351 |
| 5095_4A | *C. yungkiangensis* | 0.729246002 |
| KY406767 | *C. mairei* | 0.860944777 |
| KY406793 | *C. reticulata* | 0.804355109 |
